# Label-Free Impedimetric Biosensor Based on Molecularly Imprinted PPy/MWCNTs Nanocomposites for Sensitive and Selective Detection of *Escherichia coli*

**DOI:** 10.3390/bios16040210

**Published:** 2026-04-09

**Authors:** Wenbin Zhang, Ningran Wang, Tong Qi, Hebin Sun, Lijuan Liang, Jianlong Zhao

**Affiliations:** 1 State Key Laboratory of Transducer Technology, Shanghai Institute of Microsystem and Information Technology, Chinese Academy of Sciences, Shanghai 200050, China; 2 School of Information Science and Technology, ShanghaiTech University, Shanghai 201210, China; 3 Center of Materials Science and Optoelectronics Engineering, University of Chinese Academy of Sciences, Beijing 100049, China; 4 School of Materials Science and Engineering, Harbin Institute of Technology, Harbin 150001, China

**Keywords:** molecularly imprinted polymer, pyrrole, multi-walled carbon nanotubes, electrochemical impedance spectroscopy, *Escherichia coli*, label-free detection

## Abstract

*Escherichia coli* (*E. coli*) is a microorganism commonly found in water and food matrices, and its rapid and accurate detection is crucial for maintaining public health and ensuring food safety. However, traditional molecularly imprinted polymer (MIP) sensors often face challenges such as tedious template removal and prolonged sensing times. This study develops a label-free bacterial molecularly imprinted sensor that utilizes the synergistic effect of polypyrrole (PPy) and multi-walled carbon nanotubes (MWCNTs) to achieve highly sensitive detection of *E. coli*. Based on the large specific surface area and superior conductivity of MWCNTs, as well as the favorable electrochemical polymerization properties of PPy, a PPy/MWCNTs composite film was fabricated via a one-step electropolymerization process. The prepared sensor exhibited excellent kinetic characteristics, with a template removal time of only 15 min, and could be regenerated and used for subsequent detection within 30 min. Under optimized conditions, the biosensor showed a satisfactory linear response over the concentration range of 10^2^–10^8^ CFU/mL, with a low detection limit of 65 CFU/mL (3σ/S). Furthermore, recovery experiments conducted in tap water and lemon juice samples yielded satisfactory recoveries ranging from 87.1% to 114.8%, demonstrating the reliability and practical applicability of the proposed sensor for bacterial detection in real samples. This sensor offers advantages such as simple preparation, low material cost, and high sensitivity, providing a reliable and practical analytical platform for the rapid and reliable detection of bacteria.

## 1. Introduction

Microbial contamination in water and food is one of the major factors threatening public health and food safety [1]. *Escherichia coli*, a typical intestinal indicator bacterium, is widely distributed in the intestines of humans and animals [2]. Its detection in water and food is often regarded as an important indicator signal of fecal pollution [3]. In addition, some pathogenic *E. coli* strains can cause diarrhea, intestinal infections, and other diseases, posing a serious threat to human health. Therefore, *E. coli* is frequently employed as a model bacterium in research on pathogenic bacteria detection. Rapid, sensitive, and reliable detection of *E. coli* is of great significance for water quality safety assessment, food quality control, and environmental monitoring [4].

Currently, conventional detection methods for *E. coli* mainly include the plate culture method [5], polymerase chain reaction (PCR) [6], and enzyme-linked immunosorbent assay (ELISA) [7]. These methods have certain advantages in sensitivity and accuracy, but they generally suffer from long detection cycles, complex operation procedures, and high dependence on professional equipment and technical personnel, making it difficult to meet the requirements for on-site rapid detection and real-time monitoring. Therefore, developing a detection technology with simple operation, rapid detection, high sensitivity, and high selectivity has become an important research direction in the field of bacterial detection.

Electrochemical biosensors exhibit good application prospects in microbial detection due to their advantages, such as fast response speed, high sensitivity, and ease of miniaturization and integration [8,9,10]. Among them, electrochemical impedance spectroscopy (EIS), as a highly sensitive interface analysis technology, can achieve label-free detection of trace biological targets by monitoring the change in electron transfer impedance at the electrode interface [11,12,13]. However, the detection performance of electrochemical sensors largely depends on the electrochemical properties of the functional materials on the electrode surface and their recognition ability for target organisms. To achieve specific recognition of target analytes, various surface modification strategies have been employed, including antibody–antigen recognition [14], aptamer-based methods [15], and molecular imprinting techniques [16].

Molecular imprinting technology is a biomimetic molecular recognition strategy in which target molecules are introduced as templates during polymer formation [17,18]. After polymerization and template removal, specific recognition sites that highly match the target molecules in size, morphology, and functional group distribution are constructed on or near the surface of the material, thereby achieving selective recognition of the target molecules. Due to its highly tunable structural design and stable, reusable recognition sites, molecular imprinting technology has shown broad application potential in fields such as chemical sensing, bioanalysis, and environmental monitoring [19,20,21]. Compared with traditional biological recognition elements such as antibodies and enzymes, molecularly imprinted materials possess significant advantages such as relatively simple preparation, low cost, insensitivity to environmental conditions, and good long-term stability. Consequently, they have gradually attracted attention and found applications in the field of bacterial detection [22,23,24,25,26].

The conductive polymer polypyrrole (PPy) is widely used in the construction of electrochemical sensor functional interfaces due to its advantages such as good conductivity, biocompatibility, and the ability to achieve in situ, controlled growth via electrochemical methods [27,28,29,30]. However, pure PPy films typically have a relatively dense structure, limited specific surface area, and relatively simple electron transport pathways, and the long template removal and recognition times further limit the sensor’s applicability for on-site rapid detection [31]. Unlike graphene [32], which tends to restack and limit active surface area, or carbon quantum dots [33], which lack a continuous conductive network, multi-walled carbon nanotubes (MWCNTs) possess excellent conductivity, large specific surface area, and good mechanical stability [34,35,36,37]. Introducing MWCNTs into the PPy system can construct a three-dimensional porous conductive network structure during polymerization [38,39]. This not only significantly increases the effective reaction area of the electrode but also provides more rapid electron transport channels, thereby effectively reducing the interfacial charge transfer resistance. This composite structure not only enhances the electrochemical performance of the sensor but also facilitates the embedding of bacterial templates and the subsequent exposure of recognition sites, providing a feasible and effective material design strategy for constructing high-performance bacterial molecularly imprinted sensors.

**Scheme 1:**
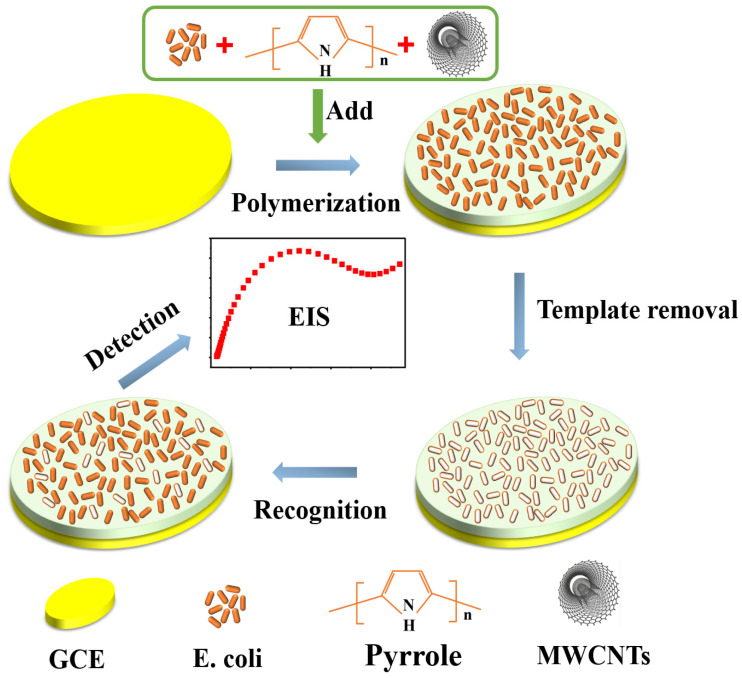
Schematic illustration for a PPy/MWCNTs-based molecularly imprinted electrochemical sensor for the detection of *Escherichia coli*.

Based on the aforementioned challenges, this study aimed to address the limitations of current bacterial MIP sensors by developing a rapid, label-free impedimetric biosensor with enhanced template removal and recognition efficiency. This study selected the commonly used and representative *E. coli* as the model bacterium and constructed a label-free impedimetric electrochemical biosensor based on a molecularly imprinted polypyrrole/multi-walled carbon nanotubes (PPy/MWCNTs) composite, as schematically illustrated in Scheme 1. A bacteria-imprinted composite film was prepared on the electrode surface via a one-step electrochemical polymerization method, ensuring a simple and controllable preparation process while introducing recognition sites with specific affinity for the target bacteria, which enable specific recognition through interactions between PPy/MWCNTs and the functional groups on the bacterial surface. Combined with electrochemical impedance spectroscopy (EIS), changes in the charge transfer behavior at the electrode interface before and after bacterial binding were monitored, thus achieving highly sensitive, label-free detection of *E. coli*. The constructed sensor demonstrated significant advantages in both fabrication simplicity and detection performance, featuring a simple preparation process, a short detection time, as well as high sensitivity and good selectivity. In particular, we significantly shortened the recognition time and template removal time, which only required 30 min and 15 min, respectively. Moreover, application validation in actual water and food samples showed that the sensor could maintain stable and reliable detection performance even under complex matrix conditions, providing a technically feasible solution with practical application potential for the rapid screening and on-site detection of bacterial contamination in water and food.

## 2. Materials and Methods

### 2.1. Reagents and Equipment

The strains used in the experiment included *Escherichia coli* (A339100), *Rhizobium* (A331025), *Saccharomyces cerevisiae* (A334031), and *Pseudomonas putida* (A333863), all purchased from Sangon Biotech (Shanghai) Co., Ltd. (Shanghai, China). The reagents used were K_3_[Fe(CN)_6_], K_4_[Fe(CN)_6_], KCl, sodium dodecyl sulfate (SDS), acetic acid (HAc), deionized water, pyrrole monomer, and multi-walled carbon nanotubes (MWCNTs), all obtained from Titan Technology Co., Ltd. (Shanghai, China). The MWCNTs had a diameter of 30–50 nm, a length of <10 µm, a purity above 95%, and were ∔OH functionalized.

Electrochemical measurements were performed using an electrochemical workstation (Gamry, Warminster, PA, USA) with a three-electrode system. The working electrode was a glassy carbon electrode (GCE, 2 mm diameter), the counter electrode was a platinum sheet, and the reference electrode was Ag/AgCl. Electrochemical impedance spectroscopy (EIS) and cyclic voltammetry (CV) tests were carried out in a 0.1 M KCl solution containing 1 mM K_3_[Fe(CN)_6_]/K_4_[Fe(CN)_6_]. A KM-MS-10 magnetic stirrer (Kemai, Ningbo, China) was used for stirring, and a constant temperature oscillator (Allsheng, Hangzhou, China) was used to assist incubation and elution. Scanning electron microscopy (SEM, Sigma 300, ZEISS, Oberkochen, Germany) was used to observe the surface morphology of different electrodes, operated at an accelerating voltage of 15.0 kV with an LED detector and a working distance (WD) of 11 mm. Elemental analysis of the electrode surface was performed using X-ray photoelectron spectroscopy (XPS, Thermo Fisher Scientific, Waltham, MA, USA) with an energy resolution of ≤0.45 eV (calibrated with the Ag 3d_5_/_2_ peak). A Fourier transform infrared spectrometer (Thermo Fisher Scientific, Waltham, MA, USA) was used for FT-IR characterization, with spectra recorded in the wavenumber range of 4000–400 cm^−1^ and at a resolution of 4 cm^−1^.

### 2.2. Bacterial Culture

Lyophilized *E. coli* strains stored at −80 °C were revived and inoculated on Luria–Bertani (LB) agar medium for culture at 37 °C. After 3–5 consecutive passages to ensure strain viability and stability, single typical colonies were selected and inoculated into 10 mL of Luria–Bertani (LB) broth, followed by overnight shaking culture at 37 °C and 200 rpm to obtain bacterial suspensions in the logarithmic growth phase. After cultivation, bacterial cells were collected by centrifugation, the supernatant was discarded, and the cells were resuspended and washed with 0.1 M KCl solution. This washing step was repeated three times to remove residual medium components. Serial dilution was used to obtain bacterial suspensions in the range of 10^2^–10^8^ CFU/mL for subsequent electrochemical detection experiments. Bacterial concentration was determined by establishing a correlation between optical density (OD_600_) and bacterial concentration (CFU/mL) using the plate counting technique. To ensure consistent sensing performance, all bacterial suspensions were utilized immediately after preparation, thereby maintaining the majority of cells in a viable and active state throughout the processing and sensing stages.

### 2.3. Electrochemical Polymerization

The glassy carbon electrode (GCE) was sequentially polished to a mirror finish using alumina powder with different particle sizes (1 μm, 0.3 μm, and 0.05 μm), and then ultrasonically cleaned in deionized water, ethanol, and a deionized water-ethanol mixture for 1 min each. After drying with nitrogen gas, the electrode was ready for use.

PPy/MWCNTs bacteria-imprinted polymers (MPBIP) were prepared via cyclic voltammetry (CV) in a solution containing 0.1 M KCl, 10^8^ CFU/mL *E. coli*, 0.05 M pyrrole monomer, and 20 μg/mL ∔OH MWCNTs. Prior to electropolymerization, the MWCNT-containing solution was ultrasonicated for 30 min to ensure homogeneous dispersion and to minimize aggregation. The scanning potential range was −0.4 V to 0.7 V, the scanning rate was 50 mV s^−1^, and the number of cycles was 15, under mild magnetic stirring. The control groups included bacteria-imprinted polymers without MWCNTs (PBIP) and non-imprinted polymers without bacterial templates (NIP). The prepared electrodes were rinsed with a large amount of deionized water to remove residual pyrrole on the electrode surface. Based on previous studies demonstrating optimal template removal using SDS/HAc solutions [29], SDS/HAc (5% *w*/*v*) was selected for the elution of the *E. coli* template. SDS, an anionic surfactant, disrupts bacterial cell membranes by solubilizing membrane lipids and denaturing proteins, while the acidic environment provided by HAc facilitates cell wall destabilization, effectively removing the bacterial templates. Meanwhile, the PPy/MWCNTs matrix remains intact due to its chemical stability under mild acidic and surfactant conditions. The electrode was immersed in 1 mL of SDS/HAc solution in a 2 mL microcentrifuge tube and oscillated at a constant speed of 400 rpm at 20 °C using a constant temperature oscillator for 15 min, followed by rinsing with deionized water.

### 2.4. Electrochemical Measurements of Bacteria

The polymerized and eluted glassy carbon electrode was incubated in 0.5 mL of 0.1 M KCl solution containing a specific concentration of *E. coli* for 30 min with constant shaking at 400 rpm. After incubation, the electrode was rinsed with deionized water. Then, CV and EIS measurements were performed in a 0.1 M KCl solution containing 1 mM K_3_[Fe(CN)_6_]/K_4_[Fe(CN)_6_]. The CV measurements were carried out in the potential range of −0.2–0.7 V at a scan rate of 50 mV s^−1^. The EIS parameters were set as follows: frequency range, 0.1–100,000 Hz; amplitude, 5 mV. The obtained Nyquist plots were fitted using Zview software (Version 2.70) based on a Randles equivalent circuit model, consisting of the solution resistance (*R*_s_), charge transfer resistance (*R*_ct_), double-layer capacitance (*C*_dl_), and Warburg impedance (*Z*_w_), to obtain the charge transfer resistance (*R*_ct_). The response capability of the sensor was expressed by Δ*R*/*R*, with the calculation formula: Δ*R*/*R* = (*R_ctB_* − *R*_ctA_)/*R*_ctA_, where *R*_ctA_ and *R*_ctB_ are the charge transfer resistance values before and after incubation, respectively.

## 3. Results and Discussion

### 3.1. Material Characterization

**Figure 1:**
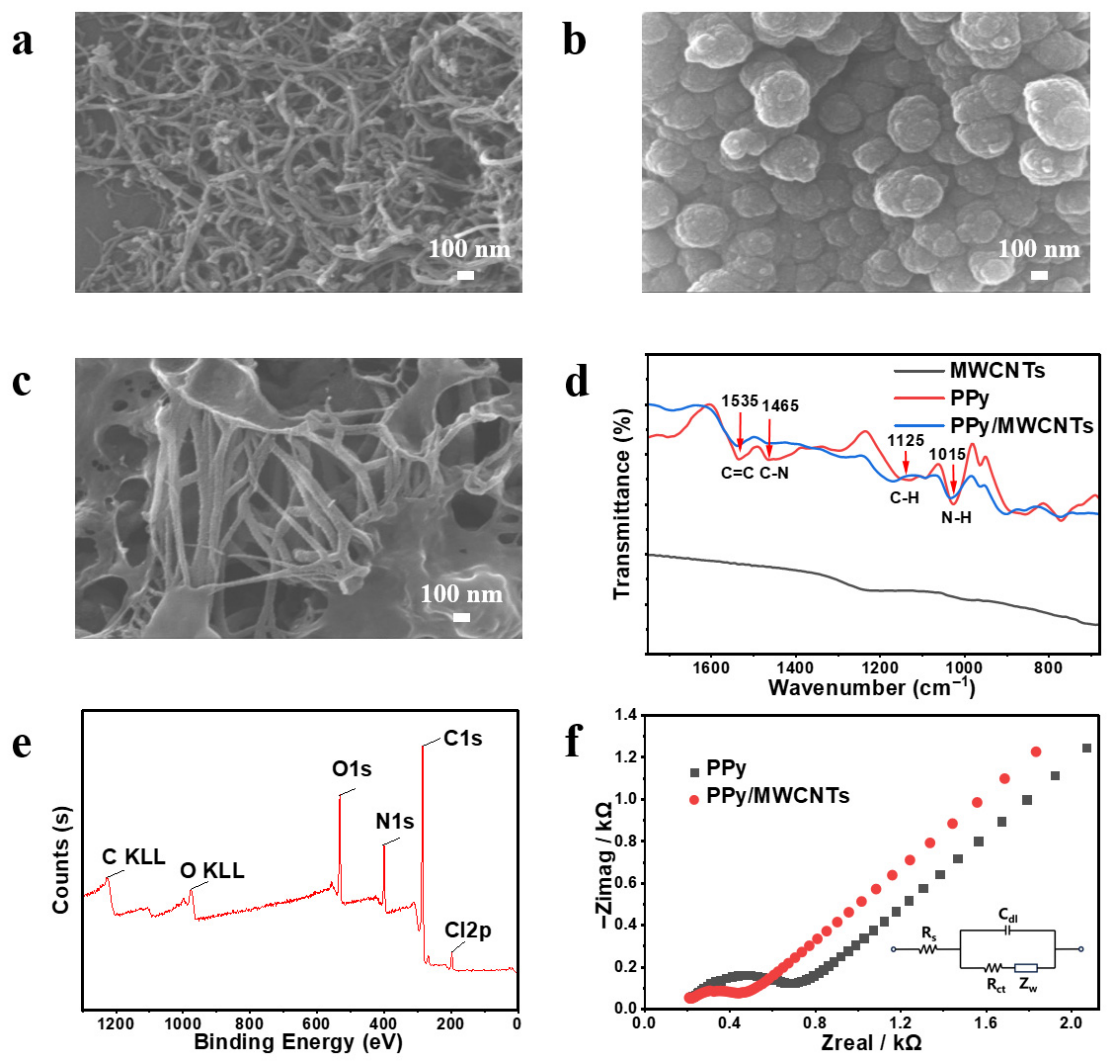
Characterization of the PPy/MWCNTs nanocomposite material. SEM images of (**a**) pristine MWCNTs, (**b**) pure PPy film, and (**c**) PPy/MWCNTs composite film; (**d**) FT-IR spectra of MWCNTs (black), PPy (red), and PPy/MWCNTs (blue); (**e**) XPS survey spectrum of the PPy/MWCNTs composite; (**f**) Nyquist plots of EIS for PPy (black) and PPy/MWCNTs (red) modified electrodes. The inset shows the Randles equivalent circuit used for fitting.

The surface morphology of multi-walled carbon nanotubes (MWCNTs), polypyrrole (PPy), and the molecularly imprinted PPy/MWCNTs composite was systematically characterized using scanning electron microscopy (SEM). As shown in Figure 1a, MWCNTs exhibited a typical hollow tubular structure, with diameters of approximately 30–50 nm and lengths extending to several micrometers, intertwined to form a loose, porous three-dimensional network skeleton, providing a good foundation for the subsequent construction of a conductive network. In contrast, the pure PPy film formed an irregular granular covering layer on the electrode surface (Figure 1b), composed of closely packed nodular structures with an average diameter of approximately 150–300 nm, with relatively limited specific surface area and structural openness. When MWCNTs were introduced into the PPy system, SEM images (Figure 1c) clearly showed the synergistic structural features of the composite: PPy coated and filled the gaps in the MWCNTs network, forming a continuous, loose three-dimensional porous composite structure. Compared to the pure PPy film, this PPy/MWCNTs composite film had higher structural openness and a larger specific surface area, providing more effective sites for bacterial template embedding and subsequent mass transfer and electron transfer during the recognition process.

FT-IR spectroscopy results (Figure 1d) further confirmed the successful composite of PPy and MWCNTs. The spectrum of MWCNTs shows a smooth curve without obvious characteristic peaks, which is consistent with the nature of carbon materials. In contrast, the spectra of PPy and PPy/MWCNTs exhibit several distinct absorption peaks. Specifically, the characteristic bands of PPy are clearly resolved at 1535, 1465, 1125, and 1015 cm^−1^, which are assigned to C=C stretching of the pyrrole ring, C-N stretching, C-H bending, and N-H deformation vibrations, respectively [40]. The characteristic vibration peaks of PPy were preserved in the PPy/MWCNTs composite, indicating that the introduction of MWCNTs did not disrupt the main chemical structure of PPy, which suggests that PPy and MWCNTs may interact mainly through π–π stacking interactions [41]. Compared to pure PPy, the intensity of some characteristic peaks in the composite slightly decreased, which might be attributed to the strong background absorption and scattering effects of MWCNTs that dominate the FT-IR spectra. XPS full-spectrum analysis (Figure 1e) confirmed the presence of carbon (C 1s), nitrogen (N 1s), and oxygen (O 1s) in the PPy/MWCNTs composite film. In addition, the Auger peaks (O KLL, C KLL) and the Cl 2p doublet peak at around 200 eV (originating from KCl present in the polymerization electrolyte) were identified. The characteristic peak of the N 1s peak at around 400 eV verified the successful synthesis of PPy, indicating that the composite material was successfully constructed on the electrode surface. Further EIS test results (Figure 1f) showed that the charge transfer resistance of the PPy/MWCNTs modified electrode was 182 Ω, which was much lower than that of the pure PPy modified electrode (483 Ω), indicating that the introduction of MWCNTs effectively reduced the interfacial electron transport resistance and improved conductivity [42], providing favorable conditions for the highly sensitive response of impedance signals in subsequent bacterial recognition processes.

### 3.2. Electrochemical Characterization

**Figure 2:**
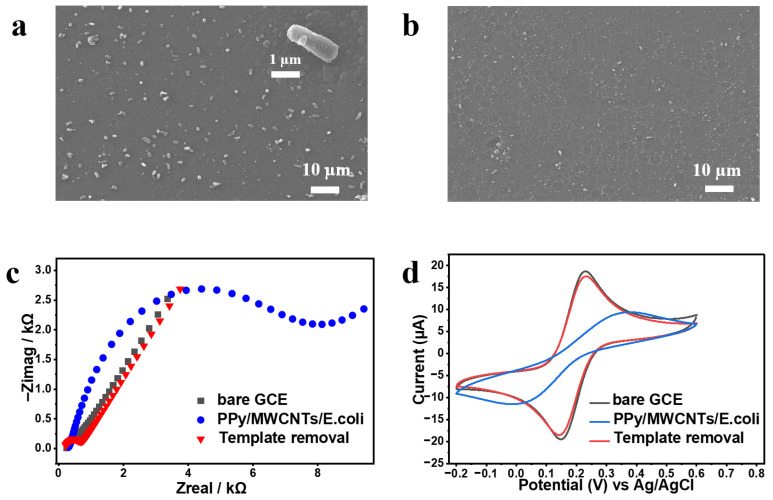
Surface characterization and electrochemical evaluation of the sensor fabrication process. (**a**) SEM image of the PPy/MWCNTs/*E. coli* electrode after electropolymerization; (**b**) SEM image of the electrode after template removal; (**c**) Nyquist plots of EIS measurements and (**d**) Cyclic voltammograms (CV) in 1 mM K_3_[Fe(CN)_6_]/K_4_[Fe(CN)_6_] containing 0.1 M KCl of the bare GCE (black), PPy/MWCNTs/*E. coli* modified electrode (blue), and after template removal (red).

During the electrochemical polymerization process containing *E. coli* templates, a large number of rod-shaped bacterial structures were observed attached to the electrode surface (Figure 2a), indicating that the template bacteria were successfully embedded into the PPy/MWCNTs composite film during polymerization. A magnified inset in Figure 2a displays a representative single bacterium with an approximate length of 2 μm and a width of 0.5 μm. Following elution treatment with SDS/HAc solution, only the PPy/MWCNTs composite structure remained on the electrode surface, with no obvious bacterial morphology observed (Figure 2b), indicating effective removal of template bacteria and the formation of recognition sites in the film capable of specifically binding target bacteria. Due to the relatively large size of the bacteria and the absence of distinct cavity structures after elution, the imprinting process is primarily governed by surface imprinting rather than bulk embedding.

The aforementioned structural changes were also reflected in the electrochemical response. As shown in Figure 2c, the bare GCE exhibited a small semicircle diameter, corresponding to a charge transfer resistance (*R*_ct_) of 108 Ω in the [Fe(CN)_6_]^3−^/^4−^ probe solution, indicating low interfacial charge transfer resistance. After embedding bacterial templates, *R*_ct_ increased dramatically to 8923 Ω due to the insulating nature of the bacteria hindering electron transfer. After template elution, *R*_ct_ decreased markedly to 492 Ω, indicating that the porous imprinted film structure was preserved. Cyclic voltammetry curves (Figure 2d) were consistent with the EIS results; the bare electrode displayed an oxidation peak current of 18.70 μA, which decreased to 9.42 μA after template embedding, and then recovered to 17.55 μA after elution, further validating the rationality of the sensor fabrication process.

### 3.3. Optimization of Experimental Conditions

To obtain the best recognition performance and stable impedance response, key parameters during sensor preparation and detection were systematically optimized, including the number of electrochemical polymerization cycles, polymerization potential, pyrrole monomer concentration, MWCNTs concentration, template removal time, and recognition time.

**Figure 3:**
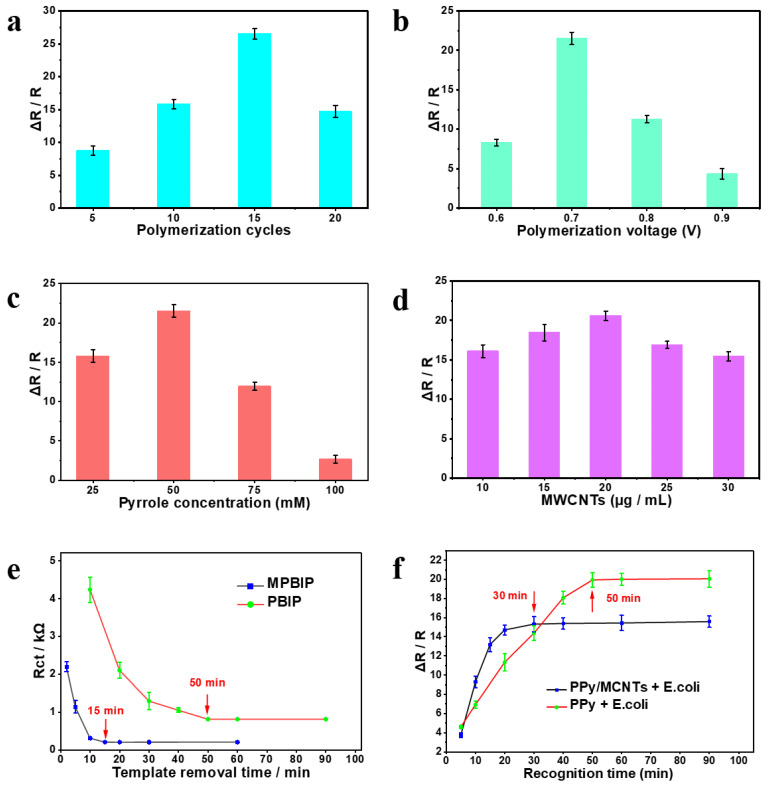
Optimization of experimental conditions for the MIP sensor fabrication and performance. (**a**) Effect of polymerization cycles; (**b**) effect of polymerization voltage; (**c**) effect of pyrrole concentration; (**d**) effect of MWCNTs concentration on the relative resistance response (ΔR/R). (**e**) Optimization of template removal time and (**f**) recognition time for MPBIP (black) and PBIP (red) sensors as measured by *R*_ct_ or ΔR/R changes. Error bars represent the standard deviation for *n* = 3.

The results showed that the number of electrochemical polymerization cycles had a significant impact on electrochemical performance of the imprinted film. With the increase in the number of cycles, the impedance response gradually increased and reached the maximum at 15 cycles (Figure 3a); a further increase in cycles led to a decrease in response due to excessive film thickness and hindered electron transport. Therefore, 15 cycles were selected as the optimal polymerization condition. The polymerization potential also affected the film structure and conductivity. A dense, uniform, and optimally electrochemically responsive imprinted film could be obtained at 0.7 V (Figure 3b), while a higher potential might cause excessive oxidation of PPy and damage the recognition performance. The optimization results of pyrrole monomer concentration showed that when the concentration was 0.05 M (Figure 3c), the prepared film achieved a good balance between structural integrity and conductivity, exhibiting the best recognition effect. In addition, an appropriate amount of MWCNTs could significantly improve the conductivity of the imprinted film and construct a three-dimensional porous network structure, thereby enhancing the impedance signal response; an excessively high concentration was prone to agglomeration, affecting the uniformity of the film. Considering both sensitivity and stability, the optimal MWCNTs doping concentration was determined to be 20 µg/mL (Figure 3d).

Meanwhile, the template removal and recognition times were optimized. As shown in Figure 3e,f, compared with the control electrode without MWCNTs, the PPy/MWCNTs composite electrode could obtain a stable and repeatable impedance response within a shorter incubation time (30 min) and elution time (15 min), which were shorter than the control group (50 min and 50 min, respectively). This performance improvement was attributed to the three-dimensional conductive network constructed by MWCNTs, which improved the electron transport efficiency and shortened the mass transfer path of bacteria in the imprinted film, thereby accelerating the recognition and dissociation processes. The above results indicated that the introduction of MWCNTs not only improved the sensitivity of the sensor but also effectively shortened the detection and regeneration time, which is beneficial for rapid detection and practical applications.

It should be noted that the optimization in this study was conducted using a one-factor-at-a-time (OFAT) approach, which does not account for potential interactions between variables. While this optimization achieved the expected results, future studies could adopt more robust strategies to make the experimental results more convincing.

### 3.4. Detection Performance

**Figure 4:**
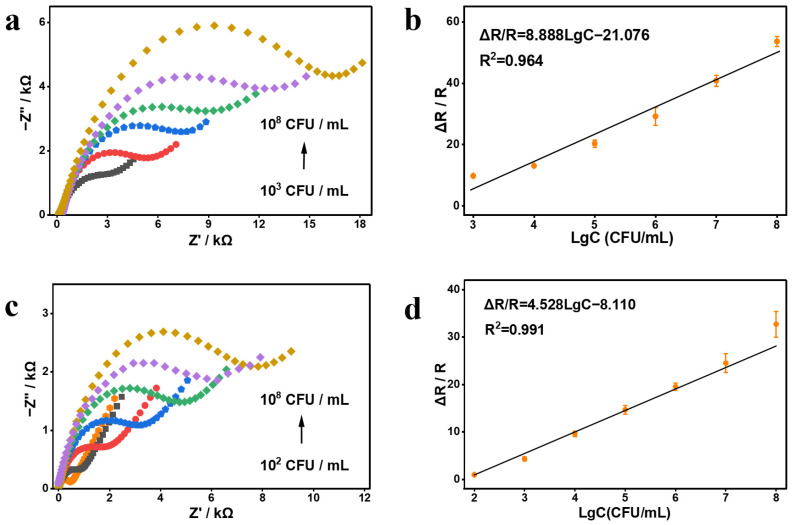
Electrochemical response and calibration curves of the sensors for *E. coli* detection at various concentrations (10^2^–10^8^ CFU/mL). (**a**) Nyquist plots and (**b**) corresponding linear calibration curve of the PBIP sensor; (**c**) Nyquist plots and (**d**) corresponding linear calibration curve of the MPBIP sensor. The relative resistance response (ΔR/R) is plotted against the logarithm of *E. coli* concentration (Lg C). Error bars represent the standard deviation for *n* = 3.

Concentration gradient response tests were performed on the sensors based on PPy and PPy/MWCNT electrodes in the range of 10^2^–10^8^ CFU/mL *E. coli*. As shown in Figure 4a,c, with the increase in *E. coli* concentration, the charge transfer resistance (*R*_ct_) of both electrodes gradually increased, indicating that bacterial binding effectively hindered interfacial electron transport. Linear fitting of Δ*R*_ct_ versus the logarithmic value of *E. coli* concentration showed that the PPy electrode exhibited a good linear relationship in the range of 10^3^–10^8^ CFU/mL, while the PPy/MWCNTs electrode showed a wider linear range 10^2^–10^8^ CFU/mL (Figure 4b,d), with linear correlation coefficients (R^2^) of 0.964 and 0.991, respectively. Compared with the single PPy electrode, the PPy/MWCNTs composite electrode exhibited better linearity and a wider linear range, indicating that the introduction of MWCNTs could effectively improve the quantitative detection performance of the sensor. Based on the 3σ/S rule [43], the LOD of the PPy/MWCNTs sensor was calculated to be 65 CFU/mL.

Table 1 summarizes the performance of previously reported molecularly imprinted sensors for bacterial detection. Compared with other reported sensors, the PPy/MWCNTs-based sensor developed in this work exhibits shorter template removal and recognition times while maintaining a wide linear detection range. Specifically, the template removal time in this work is only 15 min, which is significantly shorter than that reported for conventional pyrrole-based imprinted sensors (typically 1.5–4 h). In terms of recognition time, the proposed sensor requires only 30 min to reach equilibrium, whereas many reported systems require 1–2 h. Furthermore, the sensor maintains a wide linear detection range of 10^2^–10^8^ CFU/mL, which is comparable to or broader than most reported sensors (generally 10^1^–10^7^ or 10^3^–10^8^ CFU/mL). These results demonstrate that the introduction of MWCNTs effectively improves the detection efficiency of the molecularly imprinted electrochemical sensor.

### 3.5. Selectivity

**Figure 5:**
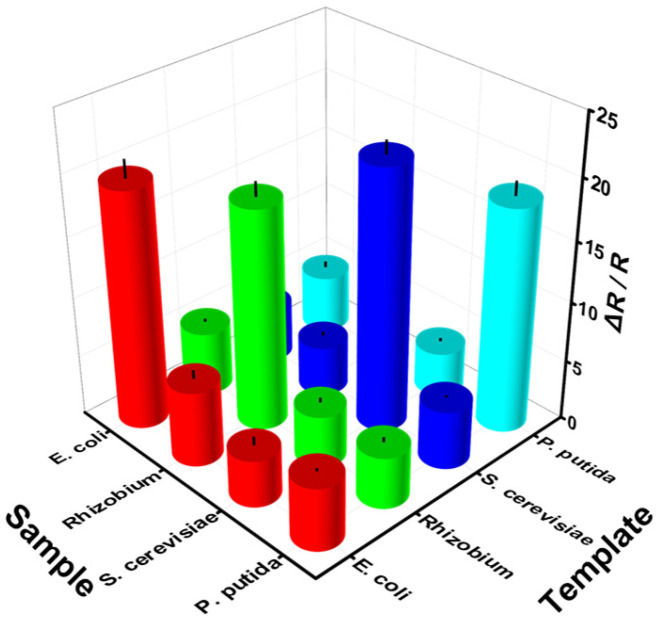
Selectivity and universality evaluation of the MIP sensors. The 3D bar chart illustrates the relative resistance responses (ΔR/R) of four MIP sensors (templated with *E. coli* (red), *Rhizobium* (green), *S. cerevisiae* (blue), and *P. putida* (cyan)) to various bacterial samples. Error bars represent the standard deviation for *n* = 3.

To evaluate the selectivity of the constructed sensor, a molecularly imprinted sensor was prepared using *E. coli* as the template bacterium, and EIS tests were performed in *E. coli* and the other three non-target microorganisms, including Gram-negative bacteria, Gram-positive bacteria, and fungi (*Pseudomonas putida*, *Rhizobium*, and Saccharomyces cerevisiae), respectively. These microorganisms were selected as representative interferents because they are commonly found in environmental and biological samples and possess different cell wall structures and surface properties, which are suitable for evaluating the specificity of the imprinted recognition sites. The results showed that the sensor exhibited the most significant impedance response to *E. coli*, which was 4–5 times higher than that toward the non-template bacteria (Figure 5). The weak response from non-target bacteria is primarily attributed to non-specific adsorption, the intensity of which is lower than that of the target. This disparity stems from the inherent differences in surface functional groups between the target and non-target species, which prevents the latter from matching the specific recognition sites on the sensor. These findings demonstrate that the PPy/MWCNTs molecularly imprinted sensor had good selectivity.

To preliminarily explore the applicability of this fabrication strategy to different bacteria, *Pseudomonas putida*, *Rhizobium*, and Saccharomyces cerevisiae were used as template bacteria, respectively, and corresponding molecularly imprinted sensors were prepared under the same electrochemical polymerization conditions. Detection of the four bacteria listed above was performed. It was found that each sensor showed a response to its corresponding template bacterium that was 3–6 times higher than the response to non-template bacteria. This result indicates that the proposed sensor fabrication strategy exhibits good applicability for the several bacterial strains tested, providing preliminary evidence that this method can be extended to the specific detection of multiple bacteria. It should be noted that the current study only tested a limited set of interferents, and mixed bacterial samples or potential chemical interferents commonly present in real-world matrices were not included. Therefore, the selectivity of this strategy in more complex matrices requires further validation.

### 3.6. Reproducibility and Stability

**Figure 6:**
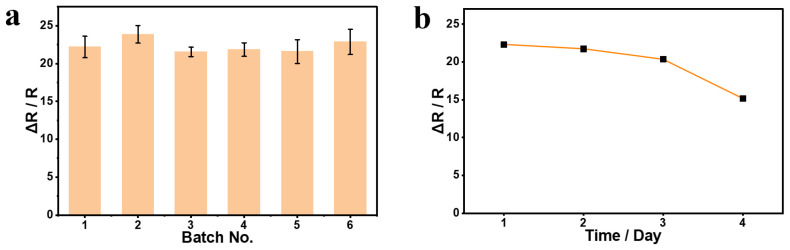
(**a**) Reproducibility; (**b**) storage stability of the fabricated electrodes.

To evaluate reproducibility, six batches of electrodes (three per batch) were prepared. After incubation in an *E. coli* suspension (1 × 10^7^ CFU/mL), electrochemical impedance spectroscopy (EIS) measurements were conducted (Figure 6a). The electrodes exhibited good intra- and inter-batch reproducibility, with maximum relative standard deviation (RSD) values of 5.45% and 3.95%, indicating reliable fabrication and sensing performance. Storage stability was further assessed by storing the electrodes under humid conditions at 4 °C. The response retained 91.2% of the initial value on day 3, remaining within an acceptable range, whereas it decreased to 67.9% on day 4, indicating a significant deterioration in recognition performance (Figure 6b). This may be attributed to the collapse of imprinted sites or deactivation of surface active sites. These results suggest that the electrodes are stable for up to 3 days under the current conditions. Further optimization of storage conditions will be pursued to extend their shelf life.

### 3.7. Real Sample Analysis

To evaluate the sensor’s performance in real samples, spike-and-recovery experiments were conducted in tap water and lemon juice, representative environmental and food matrices that simulate the sensor’s detection conditions in complex real sample environments (Figure S1). The tap water and lemon juice matrices differ in ionic strength and intrinsic pH, which may influence the electrochemical response of the sensor. The results (Table 2) showed that recoveries ranged from 87.1% to 114.8%, with relative standard deviations (RSD) of 2.34–6.50%. It should be noted that the recovery of 114.8% observed in tap water at the spike level of 1 × 10^5^ CFU/mL is slightly above 110%. While this value remains within the generally acceptable recovery range for complex matrix analysis (80–120%), it may indicate a minor positive systematic error, possibly arising from matrix-induced signal enhancement or dilution inaccuracies in the spiked bacterial suspension. Further optimization of the sample pretreatment procedure could help minimize such deviations. Overall, these results suggest that the sensor exhibits acceptable reliability and detection accuracy in real sample matrices.

## 4. Conclusions

In this study, a label-free impedimetric electrochemical biosensor based on a molecularly imprinted PPy/MWCNTs composite was successfully developed for the rapid and highly sensitive detection of *E. coli*. Experimental results demonstrated that the introduction of MWCNTs constructed a three-dimensional porous conductive network structure within the composite film, significantly increasing the electrochemically active area and electron transfer rate of the sensor. Compared to the single PPy-imprinted film, the PPy/MWCNTs composite film exhibited superior kinetic performance: template removal time was only 15 min, and a recognition time of 30 min was sufficient to reach detection equilibrium, significantly improving detection efficiency.

Under optimized conditions, the sensor showed a good impedance response to *E. coli* over a wide linear range of 10^2^–10^8^ CFU/mL, with a detection limit of 65 CFU/mL. Furthermore, cross-reactivity experiments indicated that the sensor not only exhibited high specificity for the target bacteria but also demonstrated broad universality with *Rhizobium*, *Saccharomyces cerevisiae*, and *Pseudomonas putida*, demonstrating its potential as a multi-target pathogen detection platform. Spiked recovery experiments further verified the practicality of this sensor in tap water and complex food matrices (lemon juice), with recovery rates ranging from 87.1% to 114.8%. Despite these promising results, several limitations should be acknowledged. The long-term storage stability of the imprinted electrode still requires further improvement, as the sensing performance may gradually decline during prolonged storage. In addition, the current sensing system relies on conventional electrochemical workstations, which limits its portability and on-site applicability. Future work will therefore focus on improving the structural stability of the sensing material and developing miniaturized, portable detection systems for real-time applications. Overall, this sensor offers advantages such as simple preparation, low material cost, and stable performance, providing an efficient and reliable new technological means for on-site rapid screening in environmental monitoring and food safety fields.

## Figures and Tables

**Table 1:** Performance comparison of different molecularly imprinted sensors for the recognition of bacteria.

Target Bacteria	Electrode Material	Recognition Time	Template Removal Time	Linear Range (CFU/mL)	Reference
*S. aureus*	Au/TAA	10 min	2 h	10−10^8^	[43]
*Salmonella*	Au/PDA	1 min	/	10^4^−10^7^	[44]
*Listeria*	SPCE/pyrrole	/	/	300−6700	[45]
*E. coli*	GCE/PDA/N-GQDs	/	/	10^1^−10^7^	[46]
*E. coli*	GCE/pyrrole	1 h	4 h	10^3^−10^8^	[31]
*Salmonella*	GCE/pyrrole/Mxene	1 h	5 min	10^3^−10^7^	[28]
*Vibrio parahaemolyticus*	GCE/pyrrole/D-TA	30 min	1.5 h	10^2^−10^6^	[29]
*E. coli*	GCE/pyrrole/CuPcTs	2 h	1.5 h	10^2^−10^7^	[30]
*E. coli*	GCE/pyrrole/MWCNTs	30 min	15 min	10^2^−10^8^	This work

**Table 2:** Detection results of *E. coli* in real samples.

Sample	Added (CFU/mL)	Measured (CFU/mL)	Recovery (%)	RSD (%, n = 3)
Tap water	1 × 10^3^	0.955 × 10^3^	95.5	4.06
	1 × 10^4^	0.979 × 10^4^	97.9	2.36
	1 × 10^5^	1.148 × 10^5^	114.8	2.34
Lemon juice	1 × 10^3^	0.871 × 10^3^	87.1	4.59
	1 × 10^4^	1.072 × 10^4^	107.2	6.50
	1 × 10^5^	0.891 × 10^5^	89.1	2.35

## Data Availability

The original contributions presented in this study are included in the article. Further inquiries can be directed to the corresponding author.

## Supplementary Materials

The following supporting information can be downloaded at: https://www.mdpi.com/article/10.3390/bios16040210/s1, Figure S1: Linear calibration curves of the MPBIP sensor for real samples: (a) tap water, (b) lemon juice.
