# Supplementary material for: Label-Free Impedimetric Biosensor Based on Molecularly Imprinted PPy/MWCNTs Nanocomposites for Sensitive and Selective Detection of Escherichia coli
Source: Biosensors (Basel). 2026 Apr 9;16(4):210. doi: 10.3390/bios16040210 (PMC13113960; doi:10.3390/bios16040210)
Supplement: Supplementary file 1 [file biosensors-16-00210-s001.zip › biosensors-4234006-SI.pdf]

Label-Free Impedimetric Biosensor Based on Molecularly Imprinted PPy/MWCNTs Nanocomposites for Sensitive and Selective Detection of *Escherichia coli*

Wenbin Zhang 1,2, Ningran Wang 1,2, Tong Qi 1, Hebin Sun 1, Lijuan Liang 1,3,4,* and Jianlong Zhao 1,2,3,*

1 State Key Laboratory of Transducer Technology, Shanghai Institute of Microsystem and Information Technology, Chinese Academy of Sciences, Shanghai 200050, China

2 School of Information Science and Technology, ShanghaiTech University, Shanghai 201210, China

3 Center of Materials Science and Optoelectronics Engineering, University of Chinese Academy of Sciences, Beijing 100049, China

4 School of Materials Science and Engineering, Harbin Institute of Technology, Harbin 150001, China

*Correspondence: **jlzhao@mail.sim.ac.cn; llj@mail.sim.ac.cn**

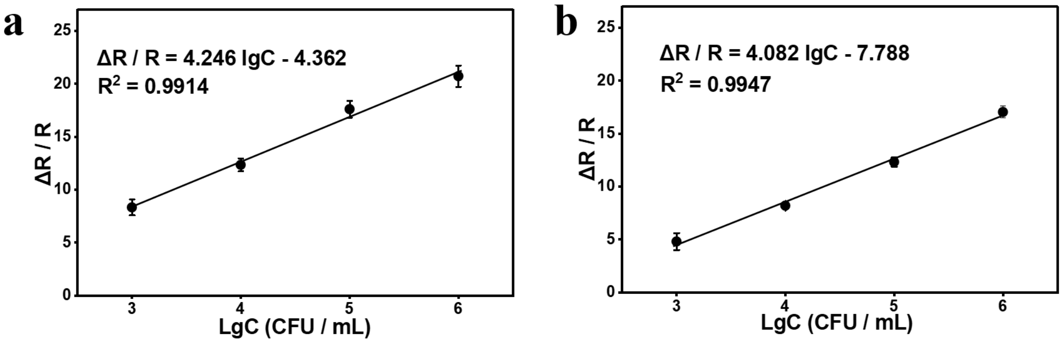

Figure S1. Linear calibration curves of the MPBIP sensor for real samples: (a) tap water, (b) lemon juice.
